# Patient-reported harm from NHS treatment or care, or the lack of access to care: a cross-sectional survey of general population prevalence, impact and responses

**DOI:** 10.1136/bmjqs-2024-017213

**Published:** 2025-04-02

**Authors:** Helen Crocker, David A Cromwell, Shivali Modha, Alastair McIntosh Gray, Chris Graham, Lavanya Thana, Raymond Fitzpatrick, Charles Vincent, Helen Hogan, Michele Peters

**Affiliations:** 1 Nuffield Department of Population Health, University of Oxford, Oxford, UK; 2 Health Services Research & Policy, London School of Hygiene & Tropical Medicine, London, UK; 3 Patient and Public Involvement and Experience Representative, London School of Hygiene & Tropical Medicine, London, UK; 4 Survey Development, Picker Institute Europe, Oxford, Oxfordshire, UK; 5 Nuffield College, University of Oxford, Oxford, UK; 6 Experimental Psychology, University of Oxford, Oxford, UK

**Keywords:** Health policy, Health services research, Patient-centred care, Patient Safety

## Abstract

**Objectives:**

The aim of this article is to provide an estimate of the proportion of the general public reporting healthcare-related harm in Great Britain, its location, impact, responses post-harm and desired reactions from healthcare providers.

**Design:**

We used a cross-sectional survey, using quota sampling.

**Setting:**

This research was conducted in Great Britain.

**Participants:**

The survey had 10 064 participants (weighted analysis).

**Results:**

In our survey 9.7% participants reported harm caused by the National Health Service (NHS) in the last 3 years through treatment or care (6.2%) or the lack of access to care (3.5%). The main location where the harm first occurred was hospitals. A total of 37.6% of participants reported a moderate impact and 44.8% a severe impact of harm. The most common response to harm was to share their experience with others (67.1%). Almost 60% sought professional advice and support, with 11.6% contacting the Patient Advice and Liaison Service (PALS). Only 17% submitted a formal complaint, and 2.1% made a claim for financial compensation. People wanted treatment or care to redress the harm (44.4%) and an explanation (34.8%). Two-thirds of those making a complaint felt it was not handled well and approximately half were satisfied with PALS. Experiences and responses differed according to sex and age (eg, women reported more harm). People with long-term illness or disability, those in lower social grades, and people in other disadvantaged groups reported higher rates and more severe impact of harm.

**Conclusions:**

We found that 9.7% of the British general population reported harm by the NHS, a higher rate than reported in two previous surveys. Our study used a broader and more inclusive definition of harm and was conducted during the COVID-19 pandemic, making comparison to previous surveys challenging. People responded to harm in different ways, such as sharing experiences with others and seeking professional advice and support. Mostly, people who were harmed wanted help to redress the harm or to gain access to the care needed. Low satisfaction with PALS and complaints services may reflect that these services do not always deliver the required support. There is a need to better understand the patient perspective following harm and for further consideration of what a person-centred approach to resolution and recovery might look like.

**WHAT IS ALREADY KNOWN ON THIS TOPIC**

- People experience physical and/or emotional harm because of their treatment or care, or the lack of access to care; however, research rarely focuses on the patient perspective, their outcomes and their responses to harm.

**WHAT THIS STUDY ADDS**

- Higher rates of harm were reported by the general population than in previous surveys, with women and socially disadvantaged people at higher risk of harm and impacted more severely by the harm.
- People mostly seek physical and emotional support after harm from family/friends or healthcare professionals to redress the harm, with men and some socially disadvantaged people less likely to seek support.

**HOW THIS STUDY MIGHT AFFECT RESEARCH, PRACTICE OR POLICY**

- Tackling the significant burden and impact of physical and emotional harm remains a priority for the NHS.
- Every person harmed should receive support from the NHS to minimise the impact of harm; however, to ensure more equitable care, particular attention should be paid to people who face barriers and those who are socially disadvantaged.

## Introduction

 Patient safety is crucial in providing high-quality health services. However, harm from system or human failure remains common. Around 10% of hospital admissions and 2–3 out of every 100 general practitioner (GP) consultations are associated with unintended and potentially harmful incidents.^1 2^ This is concerning given that healthcare-related harm can result in a range of distressing impacts across physical and/or emotional health, relationships, income and usual activities.^3^ Emotional impacts, healthcare avoidance behaviours and loss of trust in the healthcare system can persist for years.^4^

Typically, surveillance of harm and its consequences is undertaken from the clinical perspective, but this approach can miss harms that are not recorded, occur after the patient has been discharged or result from lack of access to treatment or care.^5^ Clinical record reviews are also less able to identify psychological and/or social harm. Patients are at the centre of treatment and able to observe the whole process of care. As such they are uniquely placed to fill important gaps in understanding and conceptualising harm and devising person-centred healthcare.^6^ Studies of the prevalence of harm in the general population are relatively rare. Results from a survey of residents in a region of Norway suggested 9.4% of men and 9.9% of women experienced care that led to the worsening of their health.^7^ In Great Britain, two population-based surveys identified that rates of healthcare-related harm fell from 4.8% in 2001 to 2.5% in 2013 but severity remained stable with around half reporting permanent or major disability as a result.^8^

People’s responses after harm are often attempts to mitigate and promote recovery, commonly including a search for meaning and turning to family, friends, community and health professionals for support.^9^ Healthcare professionals’ failure to listen, learn or provide necessary support are known to compound the original harm and delay recovery.^10^,^12^ Despite this awareness over many years and knowledge of the types of approaches that can promote healing, it is thought that a gap remains in the provision of supportive services.^3^ Advice and liaison services such as the Patient Advice and Liaison Service (PALS) in England were intended to offer an alternative to grievance services but remain limited in their ability to fulfil this purpose due to ambiguity in remit and limitations in availability arising from under-investment.^13^ This shortfall can lead to harmed patients taking formal action by registering a complaint or making a claim for legal damages in the hope of gaining an explanation, an apology or preventing similar problems from happening to others.^14^,^18^ Despite limited options available, few, in the end, choose formal action. A Norwegian survey found just 7% of men and 14% of women made a formal written complaint after experiencing a healthcare-related adverse event,^7^ and legal claims for financial compensation were made by just over 10% of harmed people in two population-based surveys undertaken in Great Britain in 2001 (n=8202) and 2013 (n=19 746).^8 19^

To date, most evidence on how people respond after NHS harm and underlying reasons for these responses comes from studies focused on the minority who take formal action.^14 15 17^ Few studies have identified responses after harm among the wider British population or factors associated with these responses. In addition, with long waits for treatment in the NHS, a rising number of people are experiencing harm due to failure to access care.^20^,^23^ It is not known if responses in these cases differ from those where harm is a direct consequence of treatment or care. These gaps inhibit progress in service improvements that might encourage resolution and healing, the return of trust and ultimately avoid compounding the original harm. Our study aims to provide an updated estimate of the proportion of the general public reporting healthcare-related harm from NHS treatment or care (to specifically include psychological harm and harm from the lack of access to care), the location, impact, responses and factors related to these responses.

## Methods

This cross-sectional survey was conducted with a general population sample in Great Britain as part of a mixed methods study in which in-depth qualitative interviews followed the survey.

Ethics approval was obtained from the London School of Hygiene and Tropical Medicine Ethics Committee (reference number: 23993).

### Survey design and recruitment

The 10-item questionnaire (online supplemental table S1) was developed from two previous surveys designed to collect data on adverse events and their consequences from the patient’s perspective.^8 19^ Elements from the previous questionnaire were kept, although it was updated to reflect a more current and inclusive definition of harm (ie, incorporating emotional harm and harm from the lack of access to care) and to include questions of importance to policymakers (eg, harm associated with lack of access to treatment or care and on the psychological and social impacts of harm). In line with the previous surveys, a 3 year time frame was kept aiding recall of harm and its consequences. Views on the questionnaires were elicited from NHS policymakers, patient and public involvement (PPI) representatives and the project steering committee. Amending questions meant there was less opportunity for direct comparison with the previous surveys, however, policymakers expressed a strong preference for the study to capture contemporary rates of harm based on a more inclusive and up to date definition rather than focusing on strict comparability.

The 10-item draft questionnaire was pre-tested in cognitive interviews with 11 people who had experienced harm in the NHS and one carer (see online supplemental table S2 for details of recruitment and process). Due to the COVID-19 pandemic, planned face-to-face data collection was changed to telephone interviews and the questionnaire was adapted accordingly. In a final step, Ipsos (previously Ipsos MORI), the market research agency who administered data collection, reviewed and tested the questionnaire suggesting minor changes in language and presenting response options for some questions in a random order to mitigate against biases associated with response order.

### Patient and public involvement

A PPI member (SM) is a co-applicant on the grant, a co-author and was included as a full research team member. Input was sought and received on questionnaire content and wording, recruitment processes, and reporting the findings. The study was also supported by an Expert Advisory Group (EAG) which included six diverse PPI members (in terms of age, ethnicity, religion, disability and socioeconomic status) who had experienced NHS-related harm. The EAG contributed to the design of participant information, recruitment processes and dissemination of findings.

### Data collection

Ipsos administered the survey as part of its weekly telephone omnibus using Computer Assisted Telephone Interviewing (CATI). Questions focused on whether harm was experienced in the NHS, the impact of harm, and actions taken post-harm (Box S1, online supplemental file). The Ipsos omnibus survey uses quota sampling; this is an efficient, inexpensive and widely used methodology with a good record of producing representative results. To ensure national representativeness, Ipsos sets quotas on a range of sociodemographic variables. If quotas are not achieved, data are weighted to the known sociodemographic profile of Great Britain. Based on the proportion of people reporting harm in previous surveys,^8 19^ it was estimated that 20 000 participants would be needed to identify approximately 500 people who reported harm. As a higher proportion of people reported harm in this survey, it was stopped after 10 weekly waves capturing around 1000 responses in each wave. Seven waves were conducted between November and December 2021, two in April 2022 and one in May 2022. Before the questionnaire began, a brief introduction was provided, giving people the opportunity to opt in or opt out (verbal consent). For those who opted in, standard socio-demographic information was collected (Box S1, online supplemental file).

### Statistical analysis

Data were analysed using descriptive statistics and logistic regression analysis applying the weights provided by Ipsos to ensure the sample was representative of the Great British population. SPSS version 29 and Stata version 17 were used for analysis. Statistical significance for all tests was set at 0.05. As some response categories were rarely used, the 16 ethnicity categories were reduced to six categories (online supplemental table S1). Missing data were treated as missing except for social grade where an ‘unknown’ category was used.

Participants were asked to rate the physical and emotional impact, and impact on usual activities following the harm (0 – no; 1 – mild; 2 – moderate; and 3 – severe impact). After Spearman’s rank correlation (rho) confirmed significant and moderate to high correlations between these impacts (physical and emotional r_s_=0.38, p<0.001; physical and usual activities r_s_=0.54, p<0.001; and emotional and usual activities r_s_=0.51, p<0.001), they were summed into a total impact score (0–9) and stratified into three categories (0 – no/mild 1 – moderate; and 2 – severe impact) to allow for robust analysis of the association between sociodemographic factors and responses after harm with impact. Three binary (yes/no) summary variables were created for responses following harm including: (1) sharing experiences, (2) seeking professional advice and support and (3) taking formal action. If the respondent had taken one or more actions, the summary variable was scored as ‘yes’. The Bonferroni post hoc test was used to identify group differences in impact and in post-harm responses.

Multiple logistic regression analysis was conducted with the rate of harm as the outcome variable (harmed vs not harmed) and sociodemographic factors as explanatory variables. The discrimination of the model was measured using the concordance (or c-) statistic which is a unitless index that denotes the probability that a randomly selected participant who experienced the outcome (ie, ‘experienced harm’) will have a higher modelled probability of the outcome occurring compared with a randomly selected participant who did not have the outcome (ie, ‘did not experience harm’).^24 25^ Values range from 0 to 1; a value of 0.5 indicates ‘classification no better than chance’ while a value of 1.0 indicates ‘perfect classification’. Binomial logistic regression analysis was conducted to investigate the association between sociodemographic factors and total impact score with the likelihood of sharing experiences, seeking professional advice and support or taking formal action (outcome variable).

## Results

### Participants

**Figure 1:**
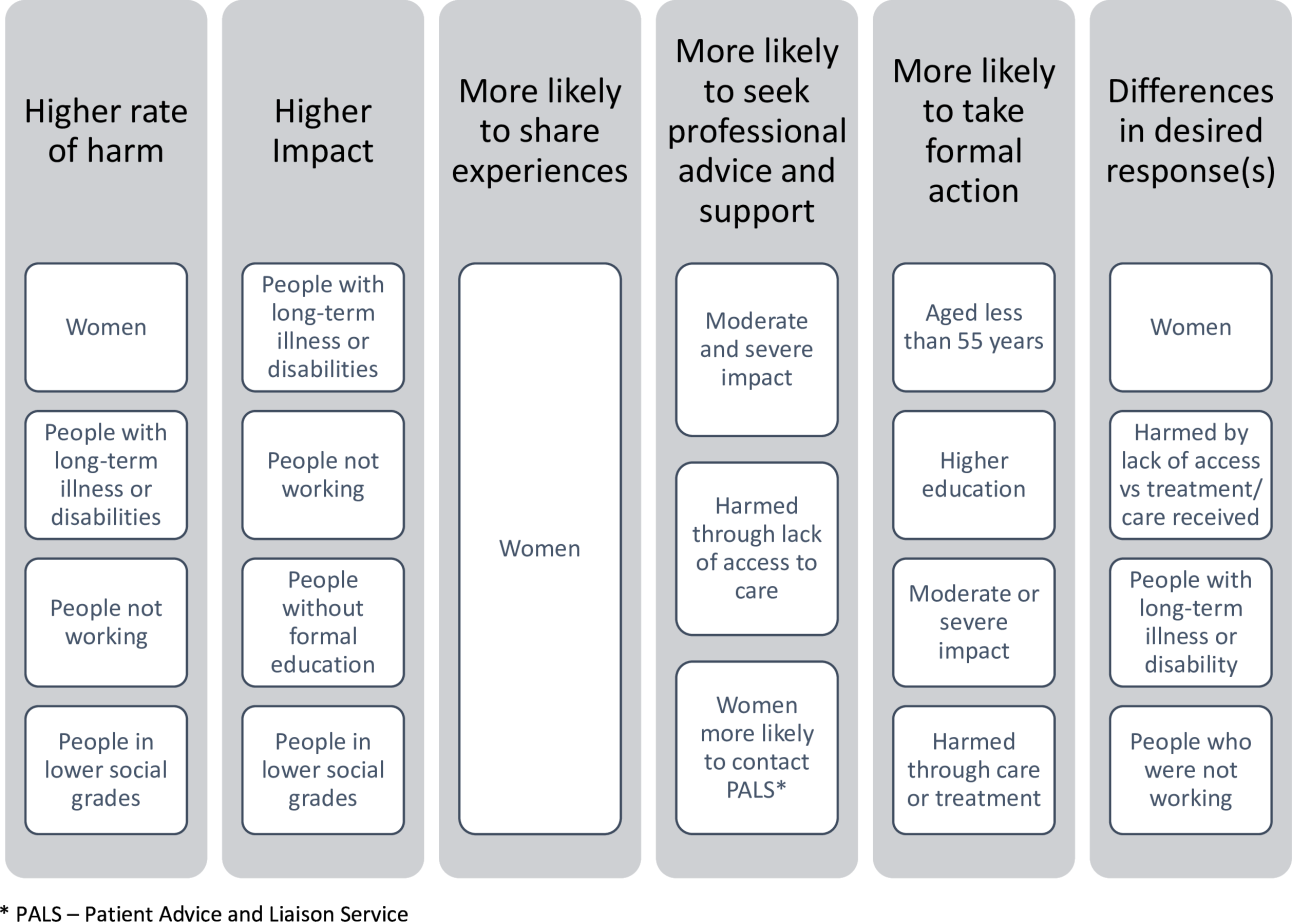
Main factors associated with experiences of harm and responses.

A total of 10 287 people participated. Exclusion of 205 participants (below 18 years (n=50), harmed from private care (n=3), ‘not sure’/refused to answer question on harm (n=152)) left 10 082 in the unweighted and 10 064 in the weighted analysis. Demographics for participants who did not report harm vs those reporting harm are shown in table 1 (demographics of the total sample are in online supplemental table S3). Some sociodemographic factors were significantly related to experiences and responses to harm; these are described below and the main findings are summarised in figure 1.

**Table 1:** Demographics for participants who did not report harm vs participants who reported harm from treatment or care received by the NHS or through lack of access over a 3 year period (weighted)

Demographics	Groups	Not harmed		Harmed		P
		n	%	n	%	
Age (years) n=10 064	18–24	1001	88.8	126	11.2	0.002
	25–34	1546	89.7	178	10.3	
	35–44	1429	89.1	175	10.9	
	45–54	1527	89.6	178	10.4	
	55–64	1386	90.2	151	9.8	
	65+	2187	92.4	180	7.6	
Sex n=9985	Male	4551	92.8	355	7.2	<0.001
	Female	4465	87.9	614	12.1	
Ethnicity n=9985	White British	7292	90.3	782	9.7	0.32
	White Other	522	88.3	69	11.7	
	Mixed	219	87.3	32	12.7	
	Asian	474	91.2	46	8.8	
	Black	386	91.7	35	8.3	
	Arab	29	87.9	4	12.1	
	Other	88	91.7	8	8.3	
Employment* n=10 027	Full-time	3657	92.9	280	7.1	<0.001
	Part-time employed	939	88.4	123	11.6	
	Self-employed	846	89.5	99	10.5	
	Economically inactive	2975	91.5	276	8.5	
	Not working (other reasons)	383	82.9	79	17.1	
	Disability or long-term illness (not working)	245	66.4	124	33.6	
Region n=10 062	East Midlands	677	91.0	67	9.0	0.016
	Eastern	890	91.8	80	8.2	
	London	1224	89.0	152	11.0	
	North East	381	90.5	40	9.5	
	North West	1041	90.7	107	9.3	
	Scotland	800	91.2	77	8.8	
	South East	1249	88.6	161	11.4	
	South West	811	91.0	80	9.0	
	Wales	417	86.9	63	13.1	
	West Midlands	799	89.6	93	10.4	
	Yorkshire and Humberside	786	92.1	67	7.9	
Education n=10 018	GCSE/O-level	1265	90.3	136	9.7	0.046
	Vocational	566	90.0	63	10.0	
	A-level or equivalent	1749	88.4	230	11.6	
	University degree or equivalent	2340	90.2	254	9.8	
	Postgraduate degree or equivalent	1172	91.6	107	8.4	
	Other	614	90.0	68	10.0	
	No formal qualifications	1329	91.4	125	8.6	
Social grade† n=9557	A	464	94.5	27	5.5	<0.001
	B	2206	92.1	172	7.9	
	C1	2239	90.4	238	9.6	
	C2	1860	90.7	191	9.3	
	D	1292	90.0	144	10.0	
	E	770	83.7	154	16.7	
	Unknown	443	87.7	62	12.3	

* Full-time - employed 30+ hours; Part-time - employed 29 hours or less; Economically inactive - full time students, still at school, retired and not working (housewife); Not working (other reasons) - unemployed and seeking work, not working for other reasons.

† Social grade: National Readership Survey Classification A (highest grade) to E (lowest grade) https://nrs.co.uk/nrs-print/lifestyle-and-classification-data/social-grade/

### Rates of harm

Out of the 10 064 participants, 9.7% (n=988) reported having been physically or emotionally harmed through NHS treatment or care, or the lack of access to care in the 3 years preceding the survey. Significantly higher rates of harm were reported by women than men (12.1% vs 7.2%, p<0.001), and significantly lower rates of harm were reported by participants aged 65 years or older (7.6%) than younger age categories (table 1). A higher proportion of participants from Wales reported harm, as did a higher proportion of those classed as social grade ‘E’ (ie, the lowest social grade). The estimated rates of harm among those who were not working due to disability or long-term illness or those not working for other reasons were considerably higher than the overall rate, at 33.6% and 17.1%, respectively. No significant differences were found in the rate of harm by ethnicity.

A regression model containing all explanatory variables was fitted to the complete cases (table 2). The adjusted odds ratios (ORs) for the various variable categories were similar in magnitude to the unadjusted values. The distribution of predicted risk of harm for the individuals who were not working differed markedly from the distributions for the other employment categories (online supplemental graph S1). The c-statistic for the model was 0.643.

**Table 2:** Associations between the risk of harm and participant characteristics, estimated using multiple logistic regression

Variable	No. of participant	% Harm	Adjusted OR	95% CI	p-value
Age group					0.045
18–24	1079	11%	1.04	0.76 to 1.44	
25–34	1679	10%	1.04	0.80 to 1.36	
35–44	1571	11%	1.02	0.78 to 1.34	
45–54 (reference category)	1674	10%	1		
55–64	1503	9%	0.76	0.58 to 0.99	
65+	2334	8%	0.69	0.50 to 0.95	
Sex					
Male (reference category)	4836	7%	1		<0.001
Female	5004	12%	1.62	1.37 to 1.91	
Ethnicity					0.52
White British (reference category)	7978	10%	1		
White Other	575	11%	1.07	0.77 to 1.48	
Mixed	246	12%	1.04	0.65 to 1.65	
Asian	511	9%	0.85	0.59 to 1.24	
Black	410	8%	0.69	0.46 to 1.05	
Other	121	9%	0.79	0.37 to 1.69	
Employment*					<0.001
Full-time (reference category)	3881	7%	1		
Part-time employed	1043	12%	1.58	1.19 to 2.09	
Self-employed	920	10%	1.65	1.26 to 2.15	
Economically inactive	3191	8%	1.43	1.10 to 1.87	
Not working: other reasons	443	17%	2.30	1.60 to 3.31	
Not working: disability / long-term illness	363	33%	5.62	3.99 to 7.91	
Government region					0.025
East Midlands (reference category)	728	9%	1		
Eastern	944	8%	0.91	0.60 to 1.36	
London	1334	11%	1.34	0.93 to 1.93	
North East	419	10%	1.11	0.68 to 1.80	
North West	1119	9%	1.08	0.74 to 1.59	
Scotland	862	9%	1.00	0.68 to 1.47	
South East	1377	11%	1.34	0.94 to 1.92	
South West	876	9%	1.02	0.69 to 1.51	
Wales	474	13%	1.57	1.06 to 2.34	
West Midlands	873	10%	1.19	0.80 to 1.75	
Yorkshire & Humberside	836	7%	0.83	0.54 to 1.27	
Education					0.54
GCSE/O-Level (reference category)	1377	10%	1		
Vocational	621	10%	1.11	0.77 to 1.60	
A-level or equivalent	1945	11%	1.26	0.96 to 1.67	
University degree or equivalent	2559	10%	1.22	0.92 to 1.60	
Postgraduate degree or equivalent	1252	8%	1.12	0.81 to 1.54	
Other	661	10%	1.12	0.77 to 1.62	
No formal education	1426	9%	0.95	0.69 to 1.30	
Social grade†					0.011
A (reference category)	484	5%	1		
B	2158	8%	1.57	0.97 to 2.52	
C1	2426	9%	1.86	1.18 to 2.94	
C2	2011	9%	1.78	1.10 to 2.90	
D	1417	10%	1.86	1.11 to 3.12	
E	888	17%	2.41	1.44 to 4.03	
Unknown	457	13%	2.31	1.39 to 3.86	

* Full-time - employed 30+ hours; Part-time - employed 29 hours or less; Economically inactive - full time students, still at school, retired and not working (housewife); Not working (other reasons) - unemployed and seeking work, not working for other reasons.

† Social grade: National Readership Survey Classification A (highest grade) to E (lowest grade) https://nrs.co.uk/nrs-print/lifestyle-and-classification-data/social-grade/

CI, confidence interval; OR, odds ratio.

### Location of harm

Of the participants who reported harm (n=988), 362 participants reported harm from a lack of access to NHS services, which corresponded to a weighted proportion of 3.5%. For the other 6.2%, the most common location where the harm first occurred was hospital, followed by GP surgery (online supplemental table S4). There were significant differences by ethnicity (p=0.016). White British people and people of mixed ethnicities were more likely to report harm through the lack of access to care whereas people of Asian, Black and ‘other’ ethnicities were more likely to report harm through NHS treatment or care received. Geographical distribution was also significantly different (p=0.034) with higher proportions of people in the East Midlands, Scotland, and Wales, and lower proportions in the North East and West Midlands reporting harm through a lack of access to treatment or care. Further information on variations in the location of harm can be found in online supplemental table S5.

### Impact of the harm

Of the participants who reported harm, 167 (17.5%) reported no /mild impact (with only six participants reporting no impact), 358 (37.6%) reported moderate impact and 427 (44.8%) reported severe impact. The impact significantly varied by employment, social grade, and level of education and by whether the harm occurred from NHS treatment or care vs the lack of access to treatment or care (online supplemental table S6). Broadly, more socially disadvantaged people were likely to report higher impact including: people who did not work due to long-term illness or disability; those not working for other reasons; those with ‘other’ or no formal education; and those in lower social grades. Some differences were substantial, for example, among those reporting harm the proportion who said that this was ‘severe’ ranged from 36.5% in social grades A & B (higher and intermediate managerial, administrative, and professional occupations) to 41.7% in grades C1 and C2 (supervisory, clerical, and junior managerial, administrative, and professional occupations, and skilled manual occupations) and 54.5% in grades D & E (semi-skilled and unskilled occupations and people who are unemployed).

### Responses after harm

Most participants who reported harm responded in multiple ways in the aftermath (table 3). Variations in the proportions of those sharing experiences, seeking professional advice and support, and/or taking formal action, stratified by demographic characteristics, impact and whether they were harmed through treatment or care vs the lack of access to care are described below (full details can be found in the online supplemental table S7).

**Table 3:** Responses after healthcare-related harm (weighted) (n=988) (Participants could select multiple responses meaning that the total number of individual actions will not add up to the total sample size of n=988)

Actions taken, grouped by category	Frequency (n)	Percent (%)
Shared experiences*	684	69.3
Spoke to friends and family	663	67.1
Shared experience on social media	93	9.4
Posted a review online	60	6.1
Seeking professional advice and support*	581	58.8
Sought support from GP surgery	343	34.7
Discussed with healthcare provider causing harm	312	31.6
Sought professional counselling	181	18.3
Contacted PALS	114	11.6
Contacted charity or advice centre	101	10.3
Took formal action*	195	19.7
Made a formal complaint	168	17.0
Contacted a solicitor	48	4.8
Made a legal claim for financial compensation	20	2.1
Contacted a claims management company	15	1.5
Other actions taken†	111	11.2
None of these	95	9.6

* Proportion of participants who scored ‘yes’ for the summary score. A ‘yes’ score was given if participants had one or more responses (within that category)

† Participants were invited to specify any other responses

PALS, Patient Advice and Liaison Service.

#### Sharing experiences

Most participants (69.3%, n=684) shared their harmful experience in one or more ways, most commonly by speaking to friends and family (67.1%, n=663). Women were significantly more likely than men to share their harmful experience. The likelihood of participants sharing their experience varied by age (p=0.009), with a greater proportion of those aged ‘35–44’ significantly more likely to share compared with participants aged ‘55–64’. Level of education was also significantly associated with sharing experiences (p=0.038), with those holding an undergraduate degree or equivalent more likely to share their experience than those with no formal qualifications. Proportions of participants sharing their experience varied by social grade (p=0.003). Regression analysis confirmed that women were more likely (OR 1.63, p=0.002) to share their experiences as were participants with social grade B (OR 5.60, p=0.027) (online supplemental table S8a).

#### Seeking professional advice and support

The majority of participants (58.8%, n=581) sought professional advice or support from one or more source(s) following their harmful experience, most commonly seeking support from their GP surgery (34.7%) or from the healthcare provider who caused the harm (31.6%). Participants harmed through lack of access to services were significantly more likely to seek professional support compared with those harmed by their treatment or care (p=0.006). The proportion of people seeking support varied by employment (p=0.020). Total impact was significantly related to seeking professional advice and support (p<0.001). Regression analysis confirmed that seeking professional advice and support was more likely in participants reporting moderate (OR 1.80, p=0.004) and severe impact (OR 1.38, p<0.001); and less likely in those harmed through treatment or care compared with those harmed through the lack of access to care (OR 0.67, p=0.01) (online supplemental table S8b). Additionally, the regression analysis showed that participants from Wales were less likely to seek professional advice and support (OR 0.40, p=0.002); whereas those who were not working (for ‘other’ reasons) and those holding undergraduate or equivalent degrees were more likely to seek support (OR 2.27, p=0.012 and OR 1.72, p=0.028 respectively).

Of those harmed, 11.6% (n=114) contacted PALS for advice and support. Women were more likely to contact PALS than men (13.3% (n=81) and 9.0% (n=32) respectively, p=0.047). Those harmed by their treatment or care were more likely to contact PALS (13.6%, n=83) than those harmed through lack of access (8.6%, n=31), p=0.018. The proportion of participants contacting PALS also varied by employment type (p=0.025), with those not working due to disability or long-term illness more likely to contact PALS than employed participants (21.3% compared with 10.0%, p<0.05). Approximately half of those who contacted PALS found the service helpful (51.8%, n=59) (online supplemental table S9) with a greater proportion of women (58.2%, n=46) finding PALS helpful compared with men (36.7%, n=11) (p=0.044).

#### Taking formal action

Relatively few people (19.7%, n=195) took formal action; 17.0% (n=168) made a formal complaint and 2.1% (n=20) made a legal claim for financial compensation. Participants more likely to take formal action were those aged ‘25–34’ (compared with those aged 65+ years), aged ‘35–44’ (compared with ages ‘55–64’ and 65+ years), those not working due to disabilities or long-term illness (compared with those employed part-time or who are economically inactive), and those harmed by their treatment or care (compared with those harmed through a lack of access to care). Those who reported severe total impact following the harm were significantly more likely to take formal action than those who reported no/mild impact (p=0.002). The majority of those who made a complaint felt that it was not handled well (63%, n=100) (online supplemental table S9).

The regression analysis confirmed that age groups 55–64, and 65+ years were less likely to take formal action (OR 0.047, p=0.038 and OR 0.31, p=0.004 respectively) and that those reporting severe impact were more likely to take formal action (OR 2.55, p<0.001) as well as those harmed through treatment or care rather than a lack of access to services (OR 2.47, p<0.001) (online supplemental table S8c). Additional groups who were more likely to take formal action were those with undergraduate and postgraduate degrees (OR 1.85, p=0.045 and OR 2.18, p=0.036 respectively) and participants with vocational qualifications (OR 2.36, p=0.031). Participants who worked part-time (OR 0.49, p=0.038) and those living in the North West (OR 0.37, p=0.03) were less likely to take formal action.

The most common reasons for not pursuing legal action were not wanting to make a financial claim against the NHS (21.6%) and not wanting financial compensation (18.2%) (online supplemental table S10). Some people (12.7%) gave an alternative reason, most commonly that there was no point, that the harm was too mild, or that nobody was to blame.

### Desired responses following harm

When people were asked what response(s) they wanted from the healthcare provider where the harm happened, participants most commonly reported that they wanted treatment or care to redress the harm (44.4%); an explanation (34.8%); and access to treatment previously refused (29.7%) (table 4).

**Table 4:** Desired reaction from healthcare provider causing harm (weighted) (participants could select multiple responses)

	Frequency (n)	Percent (%)
Treatment/care to redress harm	439	44.4
An explanation	344	34.8
Access to treatment previously refused	293	29.7
An apology	268	27.1
Staff training	247	25.0
Emotional support	217	22.0
Investigation into the causes	207	20.9
Disciplinary action	99	10.0
Financial compensation	85	8.6
None	93	9.4
Other	71	7.1
*For it not to happen again	12	1.3
*System change	25	2.6
*Acknowledgement	10	1.0

* Categories created from ‘other’ responses

There were some significant differences in the desired responses from the NHS following harm, including more women wanting an apology, an explanation, emotional support, and staff training. There were variations by age, with fewer of those aged 65+ desiring a response. A greater proportion of participants who were disabled or had long-term illnesses, or who were not working for other reasons wanted disciplinary action, an investigation, financial compensation, and an explanation into the causes of harm compared with some other employment groups. A smaller proportion of semi-skilled and unskilled manual workers (social grade D) wanted an explanation, staff training, or an investigation than those in other social grades. Those with no formal qualifications were less likely to want treatment or care to redress the harm, access to treatment previously refused, or an explanation compared with those with A levels, vocational or undergraduate qualifications. Finally, those harmed through lack of access were more likely to prioritise the need for wanted treatment or access to treatment previously refused, whereas those harmed by treatment were more likely to prioritise the need for an apology, explanation, and investigation into the causes. Details can be found in online supplemental tables S11 a,b,c).

## Discussion

A survey of over 10 000 adults in the UK found that 9.7% reported harm caused by the NHS through treatment or care (6.2%), or the lack of access to care (3.5%). The rate of patient-reported harm in this study was higher than those reported from two similar previous surveys in Great Britain (4.8% and 2.5% respectively).^8 19^ While the rate is comparable to rates reported from a Norwegian study (9.4% of men and 9.9% of women),^7^ the Norwegian study asked participants if they had ‘ever’ experienced an adverse medical event, whereas our study asked about harm over the ‘last 3 years’. Reasons for the higher rate of harm in comparison to the previous surveys in Great Britain^8 19^ are likely to be multi-factorial. First, we took a more inclusive approach to conceptualising harm by explicitly asking about emotional harm and harm through lack of access to care. The previous surveys^8 19^ asked about ‘any illness, injury or impairment’, meaning that respondents were not prompted to think about emotional harm or harm through the lack of access (see online supplemental table S1 for our and the previous question). Second, the COVID-19 pandemic was ongoing during periods of the survey and shifted NHS resources towards patients with COVID-19 as well as affecting patterns of demand and staffing levels. NHS England similarly concluded that a rise in reported patient safety incidents in 2021/2022 was at least partially related to COVID-19.^26^ Our findings point to inequities between groups of people experiencing harm, including women reporting higher rates as did socially disadvantaged people (eg, people not working due to disabilities, people in lower social grades) and older people reporting lower rates of harm. Inequities in safety have been reported previously, including higher rates of harm in women, in younger age groups, ethnic minorities, people with learning disabilities and people of lower socio-economic status.^827^,^30^ We did not find significant differences in the overall rate of harm by ethnicity, this may be due to differences in study design and setting, sampling and sample size. Additionally, our findings showed the considerable impact of harm with 44.8% reporting severe impact, with more severe impact in socially disadvantaged groups (eg, social grade or education). Little comparable evidence on self-reported impacts is available. Fenn *et al,*^19^ and Grey *et al,*^8^ found that 44–50% of harmed participants reported permanent or major disability; however comparability to our findings is limited as they predominantly focused on physical impact.

People respond to NHS harm, in a variety of ways and most took multiple actions. It was common to share experience with friends and family or seek professional advice and support. Far fewer took formal action. Similar to previous studies, greater impact of harm was associated with a higher likelihood of seeking professional support and advice and taking formal action indicating an increased need for support to redress the harm and reduce its long-term effects.^31 32^ The proportions of participants desiring an apology or explanation, or financial compensation were comparable to that found in the two previous surveys.^8 19^ Our survey found a larger proportion of people expressing a preference for support to deal with the consequences of harm (44.4% for treatment or care and 22.0% for emotional support) compared with Gray *et al*, (12.0%).^8^ Although significantly more of those who were harmed through lack of access to care wanted treatment or care to redress harm (53.6% vs 39.6%), these proportions were also substantial in those harmed through their treatment or care. Older people, men, those with lower educational achievement or low socioeconomic grouping were less likely to share their experiences and older people were less likely to make a formal complaint.

Findings confirm that the majority of people who report harm actively seek resolution and recovery through multiple types of actions, although many of their responses are not visible in statistics on complaints. Our observation that nearly 60% make informal approaches to healthcare providers or relevant support services, mirrors those from a previous UK survey where 38% of those unhappy with their care discussed this with a healthcare professional.^33^ Our findings show variation in who seeks professional support is mostly driven by the impact of the harm and by harm due to a lack of access to care. Taking such a step is not easy and may come with fears of being labelled troublesome.^34^ Informal patient-provider contact represents an opportunity for sharing experiences, gaining information and advice, and discussing needs. Moore *et al,* highlight how important it is that affected individuals and families feel their experiences and the consequences are understood.^35^ Such interactions afford the provider a chance to signpost or make a referral to relevant sources of support tailored to needs. Effective use of these early contacts may mitigate against the long-term consequences of loss of trust and altered service use that can occur in the aftermath of harm.^4 36^ To facilitate meaningful conversations, NHS staff would need to feel comfortable encouraging people to talk about their healthcare experiences and in accepting negative feedback in a constructive way. This is likely to require a substantial cultural shift.^37^,^40^

The majority (63%) of those who made a formal complaint felt that it was not handled well. Impersonal, dismissive or defensive responses that do not meet needs have been shown to cause psychological distress.^41^ When people cannot get validation of their experiences and adequate help with recovery through these routes, they may be forced to consider taking legal action. Around 50% of those who make claims for financial compensation will have previously submitted a written complaint, suggesting that shortcomings in the management of concerns at this level can have a direct impact on the NHS litigation bill.^42^ Gray *et al*, found a higher litigation rate than this survey (10% vs 2.1%).^8^ This may be due to our wider definition of harm or may be a reflection of the trend in the reduction of volume of clinical negligence claims as alternative approaches to settling such grievances have been instituted.^43^ Loyalty to the NHS remains present, with 21.6% of harmed participants indicating that they would not want to make a financial claim against the NHS. Our study found that few people (more likely to be female) contacted PALS and many that did were not helped, reflecting previously highlighted deficiencies with the service.^13^

These findings shed light on the types of services that might be required to address the gap in provision of information after harm and support with recovery. Over 40% of people desired treatment or care to address physical or psychological needs (with higher proportions of those reporting harm through care or treatment seeking this type of response) and such support should be seen as an important aspect of any holistic provision. For some people, a trusted healthcare professional may be the right person to provide early validation, advice and support, and for others, independent advocacy may be more helpful. We found differential rates of harm and uptake of grievance services across a range of background variables. Previous studies confirm that older people, those with a lower educational level or on a lower income are less likely to complain.^31 44^ Services supporting recovery after harm would need to identify populations most at risk and address any potential barriers to access for such groups. This may only be achieved by proactively identifying those harmed and offering support.^45 46^

This study is one of the first to explicitly assess actions after harm due to lack of access to care. A recent survey found that around half of those waiting for treatment were suffering physical and psychological consequences and that the distribution of waiting was not even, with more people living in deprived areas waiting longer.^22^ An important finding of this study is that people harmed through a lack of access to care also require support, and the responses they desire differ from people who were harmed through treatment of care received. Having their situation recognised and being signposted towards appropriate support via a local healthcare provider may be vital in reducing harm in this group.

### Limitations

There are some limitations to this research. The survey was conducted during the COVID-19 pandemic, which may have led to a higher rate of harm than usual and makes findings less comparable to other studies. The COVID-19 pandemic started in late 2019, with the first national UK lockdown starting March 2020 and the majority of restrictions ending March 2022. The survey data were collected between November 2021 and May 2022, and asked participants to report on a 3 year timeframe (ie, the range of November 2018 to May 2022). Moreover, changes to the survey questionnaire limit comparability with the results of similar 2001 and 2013 surveys: unlike in previous studies, we prompted respondents to consider emotional harm and harm arising from a lack of access to care, which widens the scope and will have contributed to higher rates of reported harm.

The use of quota sampling is another potential limitation. Quota sampling is a convenience method that does not meet the basic requirements of randomness present in statistical sample surveys and there may be issues of representativeness and bias within.^47^ Studies have found inconsistent results when comparing quota and probability sampling methods; some report that quota methods are an acceptable alternative to probability sampling^48^ while others are more critical.^49^ However, quota sampling is widely used in market and public opinion research because it is cheaper and less burdensome than other methods. Biases arising from sampling errors are likely to be small especially when taking into account the impact of non-response in probability sample surveys.^47 48^

Also, the use of a survey methodology means that depth and nuance cannot be captured, which may be particularly important in less explored areas such as the lack of access to treatment. Qualitative methods would allow further exploration of topics that currently have limited evidence. In addition, the overall sample size was large which may have impacted on how many significant associations were found. However, for the analyses of those who reported harm, sample sizes were smaller, including some too small for statistical analysis, for example ethnicity categories needed to be collapsed. Even at the collapsed level, the numbers in some ethnic minority categories were too small for regression analysis, which meant we were limited in the extent to which we could explore known inequities in ethnic minority groups. A larger scale study, or one targeted specifically at ethnic minority groups would be needed to provide robust enough sample sizes for meaningful analysis. Surveys of the general public have the potential to reach both current service users and those who no longer use services as a result of poor outcomes. Repeating such surveys will allow the identification of trends, particularly for relatively under-researched areas such as harm caused through lack of access to care, as well as aiding the evaluation of policy initiatives.

## Conclusion

Surveys of the general public contribute to a whole system view of safety. This study found higher rates of NHS-related harm than previous surveys and showed the impact is likely to have significant consequences for individual patients, families and carers, health services and the economy. People responded to healthcare-related harm in multiple different ways. The majority shared their experiences with others and made contact with health service staff seeking explanations and support. The study highlights many instances of significant inequities in rates and impact of harm, as well as in responses in the aftermath of harm. Taken together, the differences point towards socially disadvantaged people being more likely to be harmed, have higher impact and to be less able to advocate for themselves in the aftermath of harm (figure 1). However, some (not necessarily disadvantaged) groups also face differences (eg, women report higher levels of harm) and barriers in getting support such as those where the harm had a mild impact and men being less likely to seek support. While all people who have experienced harm should be supported meaningfully by healthcare services to aid their recovery, most health systems, including the NHS, are advocating for a focus on inequalities. This could be done through for example, greater patient, family and carer involvement following a safety incident;^50^ giving greater consideration to how a patient’s sociodemographic background influences risk of harm; or supporting diverse leadership in healthcare that mobilises the system towards meaningful action.^51^ In line with this, frameworks are being proposed to support and promote the health of the most vulnerable^52^ or an inclusive health service targeting the reduction of inequities.^53 54^ Interest has also been growing in adopting trauma informed practice as this approach enhances access for all and in particular those who find it harder to get support.^55^ Adopting patient-centred and inclusive services that positively support the most disadvantaged or vulnerable may lead to systems that are better enabled to protect all people from healthcare harm, and to support people in their recovery when they are harmed.

## Supplementary material

10.1136/bmjqs-2024-017213 online supplemental file 1

## Data Availability

Data are available upon reasonable request.
