# Supplementary material for: Patient-reported harm from NHS treatment or care, or the lack of access to care: a cross-sectional survey of general population prevalence, impact and responses
Source: BMJ Qual Saf. 2025 Apr 2;35(1):e017213. doi: 10.1136/bmjqs-2024-017213 (PMC12772557; doi:10.1136/bmjqs-2024-017213)
Supplement: online supplemental file 1 [file bmjqs-35-1-s001.pdf]

Supplementary file: Patient-reported harm from NHS treatment or care, or the lack of access to care

Table S1: Survey questions

Question relating to harm

- Q1. In the last three years, have you been physically or emotionally harmed by your NHS treatment or care, or the lack of it? [1]
- Q2. Where did the harm first happen?
- Q3. … what impact, if any, did the harm have on your physical health?
- Q4. … what impact, if any, did the harm have on your emotional well-being or mental health?
- Q5. … what impact, if any, did the harm have on your usual activities (for example, work, education, social activities, or caring responsibilities)?
- Q6. Which of the following actions, if any, did you take as a result of being harmed?
- Q7. How well, or not, did you think your formal complaint was handled?
- Q8. How helpful, or not, did you find PALS, the Patient Advice and Liaison Service?
- Q9. … what response did you want from the healthcare provider where the harm happened?
- Q10. What is the main reason, if any at all, you didn't pursue a legal claim for financial compensation?

Demographics

- Age (18-24; 25-34; 35-44; 45-54; 55-64; 7: 65+
- Sex (male and female)
- Geographical region (East Midlands; Eastern; London; North East; North West; Scotland; South East; South West; Wales; West Midlands; and Yorkshire and Humber)
- Employment (Full-time employed; Part-time employed; Self-employed; Economically inactive (students, retired people, home keeper); Not working (unemployed and seeking work, not in paid work for other reasons); Disabled/ long-term illness (not working).
- Self-reported ethnicity (White British; White Other; Mixed; Asian/ Asian British, Black/ Black British; and Other) [2]
- Social grade - National Readership Survey Classification A (highest grade) to E (lowest grade) https://nrs.co.uk/nrs-print/lifestyle-and-classification-data/social-grade/

[1] The previous question on the rate of harm (Gray et al (8) and Fenn et al (19)) was "Over the last three years, have you suffered any illness, injury or impairment that in your opinion was caused by your medical treatment or care?"

[2] Categories computed from 16 response options used by IPSOS and by the Office of National Statistics. The 16 response options are: White British ('White British); White Irish, White Gypsy /Traveller and White Other ('White Other'); Mixed White/Black Caribbean, Mixed White/Black African, Mixed White And Asian and Mixed Other ('Mixed'); Asian Indian, Asian Pakistani, Asian Bangladeshi, Asian Chinese, and Asian Other (Asian/ Asian British); Black African, Black Caribbean, and Black Other (Black/ Black British); Arab and Other (Other)

Supplementary file: Patient-reported harm from NHS treatment or care, or the lack of access to care

Table S2: Cognitive Interviews

**Recruitment**
Cognitive interviews, which were conducted over the telephone with eight women and four men (age range 47-73 years; 10 White British; 2 working, 3 retired, 3 permanently sick or disabled, 1 doing something else and 3 unknown); and 11 of whom had experience of harm and 1 was the spouse of someone with experience of harm. The 12 participants were recruited via two patient and public involvement (PPI) groups and snowballing. People were eligible to participate if they were aged ≥18 years and reported physical or emotional harm through the NHS.
**Process**
A concurrent probing method,[1] was used to evaluate the questionnaire, with a researcher reading each question to the participant and the participant providing their response. This was followed by a series of cognitive probes to assess comprehension, ability to retrieve the requested information, certainty of response, and the suitability and breadth of response options. The interviews were audio-recorded. Findings for each round were summarised in a matrix, discussed by the research team. Where necessary, revisions to a question were made prior to the next round. After three rounds, key issues had been identified and rectified.

[1] Willis, G.B. Analysis of the cognitive interview in questionnaire design. New York: Oxford University Press

Supplementary file: Patient-reported harm from NHS treatment or care, or the lack of access to care

Table S3: Sociodemographic factors for the total sample (n=10,064) (weighted analysis)

		Total n	%
**Demographics**	Groups		
**Age (years)**	18-24	1126	11.2
	25-34	1725	17.1
	35-44	1604	15.9
	45-54	1705	16.9
	55-64	1537	15.3
	65+	2367	23.5
**Sex**	Male	4906	48.8
	Female	5079	50.5
**Ethnicity**	White British	8074	80.2
	White Other	591	5.9
	Mixed	251	2.5
	Asian	520	5.2
	Black	421	4.2
	Other	128	1.3
**Employment** [1]	Full-time	3937	39.3
	Part-time employed	1062	10.6
	Self-employed	945	9.4
	Economically inactive	3251	32.4
	Not working	463	4.6
	Disability or long-term illness	369	3.7
**Region**	East Midlands	744	7.4
	Eastern	970	9.6
	London	1376	13.7
	North East	421	4.2
	North West	1148	11.4
	Scotland	876	8.7
	South East	1410	14.0
	South West	891	8.9
	Wales	480	4.8
	West Midlands	892	8.9
	Yorkshire and Humberside	853	8.5
**Education**	GCSE/O-level	1402	13.9
	Vocational	629	6.3
	A-level or equivalent	1979	19.8
	University degree or equivalent	2595	25.9
	Postgraduate degree or equivalent	1280	12.8
	Other	683	6.8
	No formal qualifications	1454	14.5

Table S3 (continued): Sociodemographic factors for the total sample (n=10,064) (weighted analysis)

		Total n	%
**Social grade [2]**	A	492	4.9
	B	2178	21.6
	C1	2477	24.6
	C2	2052	20.4
	D	1436	14.3
	E	925	9.2
	Unknown	506	5.0

[1] Full-time – employed 30+ hours; Part-time – employed 29 hours or less; Economically inactive – full time students, still at school, retired or not in paid work due to long-term illness or disability; Not working – Unemployed and seeking work, Not in paid work for other reasons, or not working – housewife

[2] Social grade: National Readership Survey Classification A (highest grade) to E (lowest grade) https://nrs.co.uk/nrs-print/lifestyle-and-classification-data/social-grade/

Supplementary file: Patient-reported harm from NHS treatment or care, or the lack of access to care

Graph S1: Distribution of predicted risk of harm from the full model, stratified by employment categories.

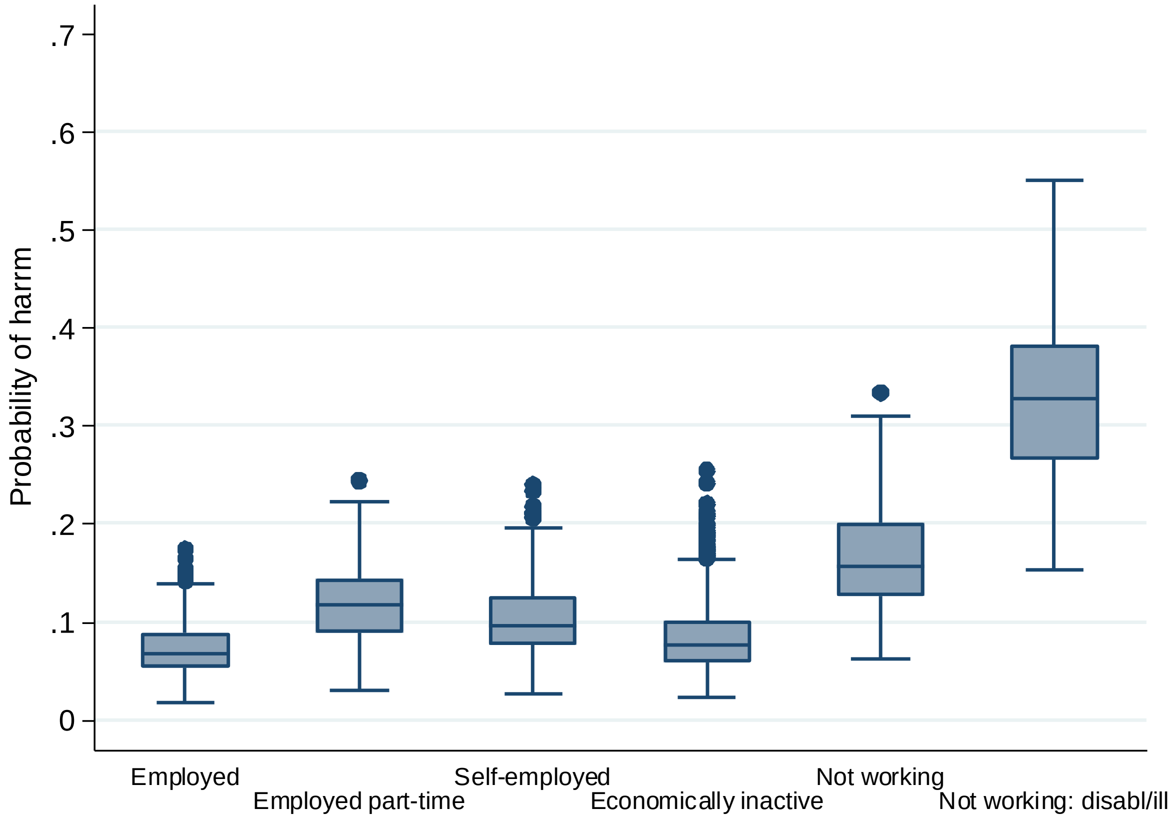

Key: Disabl/ill = Not working due to disability or long-term illness

Supplementary file: Patient-reported harm from NHS treatment or care, or the lack of access to care

Table S4: Location where the harm first occurred

	n	%
**Lack of access to care**	362	36.7
**Hospital – all**	274	27.7
Hospital – inpatient	120	12.2
Hospital - outpatient	92	9.3
Hospital - day surgery	37	3.7
Hospital - Other	25	2.5
**GP surgery**	205	20.7
**Home**	61	6.2
**Other**	52	5.3
**Dental surgery**	22	2.2

Supplementary file: Patient-reported harm from NHS treatment or care, or the lack of access to care

Table S5: Socio-demographics variables for those harmed through lack of access compared to those harmed through treatment or care received (weighted analysis) (total n =988)

		Harmed through lack of access		Harmed through treatment/ care received		p
		n	%	n	%	
**Demographics**	Groups					
**Age (years) (n=976)**	18-24	48	13.3	77	12.5	0.37
	25-34	56	15.5	118	19.2	
	35-44	61	16.9	113	18.4	
	45-54	62	17.1	114	18.6	
	55-64	58	16.0	90	14.7	
	65+	77	21.3	102	16.6	
**Sex (n=958)**	Male	138	39.1	214	35.4	0.25
	Female	215	60.9	391	64.6	
**Ethnicity (n=978)**	White British	297	83.0	476	78.4	**0.016**
	White Other	25	7.0	44	7.2	
	Mixed	16	4.5	15	2.5	
	Asian	12	3.4	34	5.6	
	Black	8	2.2	26	4.3	
	Other	0	0	12	2.0	
**Employment [1] (n=973)**	Full-time	102	28.2	176	28.8	0.30
	Part-time employed	54	14.9	68	11.1	
	Self-employed	34	9.4	64	10.5	
	Economically inactive	108	29.8	168	27.5	
	Not working	27	35.5	86	69.9	
	Disability / long-term illness	37	30.1	86	69.9	

Table S5 (continued): Socio-demographics variables for those harmed through lack of access compared to those harmed through treatment or care received (weighted analysis) (total n =988)

		Harmed through lack of access		Harmed through treatment/ care received		p
		n	%	n	%	
**Region (n=978)**	East Midlands	17	4.7	50	8.1	**0.034**
	Eastern	31	8.5	49	8.0	
	London	56	15.4	94	15.3	
	North East	9	2.5	31	5.0	
	North West	34	9.4	70	11.4	
	Scotland	34	9.4	42	6.8	
	South East	56	15.4	105	17.1	
	South West	37	10.2	42	6.8	
	Wales	32	8.8	31	5.0	
	West Midlands	31	8.5	60	9.8	
	Yorkshire and Humberside	26	7.2	41	6.7	
**Education**	GCSE/O-level	43	11.9	93	15.2	**0.004**
	Vocational	22	6.1	41	6.7	
	A-level or equivalent	68	18.8	160	26.1	
	University degree or equivalent	115	31.9	136	22.2	
	Postgraduate degree or equivalent	33	9.1	73	11.9	
	Other	30	8.3	37	6.0	
	No formal qualifications	50	13.9	72	11.8	
**Social grade [2]**	A	22	36.7	38	63.3	0.72
	B	7	25.9	20	74.1	
	C1	62	36.5	108	63.5	
	C2	88	37.3	148	62.7	
	D	67	36.0	119	64.0	
	E	61	42.7	82	57.3	
	Unknown	54	35.3	99	64.7	

[1] Full-time – employed 30+ hours; Part-time – employed 29 hours or less; Economically inactive – full time students, still at school, retired or not in paid work due to long-term illness or disability; Not working – Unemployed and seeking work, Not in paid work for other reasons, or not working – housewife.

[2] Social grade: National Readership Survey Classification A (highest grade) to E (lowest grade) https://nrs.co.uk/nrs-print/lifestyle-and-classification-data/social-grade/

Supplementary file: Patient-reported harm from NHS treatment or care, or the lack of access to care

Table S6: Differences in total impact by socio-demographics and harm through access to treatment or care versus lack of access to treatment or care (* only significant results are reported for relevant post-hoc analyses)

		No/mild		Moderate		Severe			
		**N**	**%**	**N**	**%**	**N**	**%**	**p**	**Post hoc analysis ***
**Age**	*18-24*	21	17.1	55	44.7	47	38.2	0.49	--
	25-34	36	20.5	68	38.6	72	40.9		
	35-44	27	16.0	65	38.5	77	45.6		
	45-54	26	15.5	63	37.5	79	47.0		
	55-64	24	16.8	44	30.8	75	52.4		
	65+	34	19.5	63	36.2	77	44.3		
**Sex**	*Male*	67	19.6	127	37.2	147	43.1	0.53	N/A
	Female	99	16.7	226	38.2	353	37.8		
**Ethnicity**	*White British*	130	17.2	276	36.6	348	46.2	0.74	--
	White Other	16	23.9	26	38.8	25	37.3		
	Mixed	4	13.3	14	46.7	12	40.0		
	Asian	9	19.6	20	43.5	17	37.0		
	Black	5	14.7	14	41.2	15	44.1		
	Other	2	20.0	2	20.0	6	60.0		
**Employment** [1]	*Employed*	57	21.2	120	44.6	92	34.2	**<0.001**	No/ mild impact: Employed, self-employed, economically inactive vs. disability/ long-term illness; Moderate impact: Employed, employed part-time; self-employed and economically inactive vs. disability/long-term illness; High impact: Employed vs not working, and employed vs disability/ long-term illness.
	Employed part-time	18	15.4	52	44.4	47	40.2		
	Self-employed	22	22.4	37	37.8	39	39.8		
	Economically inactive	52	19.5	98	36.8	116	43.6		
	Not working	11	14.1	25	32.1	42	53.8		
	Disability / long-term illness	7	5.8	23	19.2	90	75.0		
**Region**	*East Midlands*	10	15.4	21	32.3	34	52.3	0.42	--
	Eastern	22	29.3	25	33.3	28	37.3		
	London	23	15.6	65	44.2	59	40.1		
	North East	7	18.9	16	43.2	14	37.8		
	North West	15	14.7	40	39.2	47	46.1		
	Scotland	10	13.2	27	35.5	39	51.3		
	South East	28	17.5	59	36.9	73	45.6		
	South West	14	17.9	26	33.3	38	48.7		
	Wales	9	15.0	17	28.3	34	56.7		
	West Midlands	18	20.9	35	40.7	33	38.4		
	Yorkshire & Humberside	12	18.2	27	40.9	27	40.9		

Table S6 (continued): Differences in total impact by socio-demographics and harm through access to treatment/care versus lack of access to treatment or care (* only significant results are reported for relevant post-hoc analyses)

		No/mild		Moderate		Severe			
		**N**	**%**	**N**	**%**	**N**	**%**	**p**	**Post hoc analysis ***
**Education**	GCSE/O-Level	24	18.2	53	40.2	55	41.7	**<0.001**	No/mild: A-level vs Postgraduate degree<br>Moderate: University degree vs No formal education<br>Severe: University degree and Postgraduate degree vs No formal education
	Vocational	8	13.1	22	36.1	31	50.8		
	A-level or equivalent	30	13.5	87	39.0	106	47.5		
	University degree or equivalent	42	17.3	111	45.7	90	37.0		
	Postgraduate degree or equivalent	30	29.1	35	34.0	38	36.9		
	Other	10	15.4	19	29.2	36	55.4		
	No formal education	20	17.1	29	24.8	68	58.1		
**Social grade** [2]	*A*	4	14.8	11	40.7	12	44.4	**0.005**	Severe impact:<br>A vs B, D, E, Unknown vs B, D, E
	B	32	19.4	75	45.5	58	35.2		
	C1	39	17.0	96	41.9	94	41.0		
	C2	40	22.3	63	35.2	76	42.5		
	D	16	11.6	48	34.8	74	53.6		
	E	19	12.5	49	53.6	84	55.3		
	Unknown	16	26.2	17	27.9	28	44.8		
**Harmed through treatment or care vs lack of access**	Treatment or care	120	20.1	216	36.2	260	43.6	**0.019**	N/A
	Lack of access	45	13.0	141	40.6	161	46.4		

[1] Full-time – employed 30+ hours; Part-time – employed 29 hours or less; Economically inactive – full time students, still at school, retired or not in paid work due to long-term illness or disability; Not working – Unemployed and seeking work, Not in paid work for other reasons, or not working – housewife.

[2] Social grade: National Readership Survey Classification A (highest grade) to E (lowest grade) https://nrs.co.uk/nrs-print/lifestyle-and-classification-data/social-grade/

Supplementary file: Patient-reported harm from NHS treatment or care, or the lack of access to care

Table S7. Proportion of participants reporting responses and differences by socio-demographic variables and access to treatment (weighted) (* Post-hoc analyses are shown only for significant pairwise comparisons, p<0.05)

	Sharing experiences				Seeking professional advice and support				Making a formal complaint or taking legal action			
**Sex**	**n**	**%**	**p**	**Post-hoc***	**n**	**%**	**p**	**Post-hoc***	**n**	**%**	**p**	**Post-hoc***
Male	220	61.8	<0.001	--	212	59.7	0.63	--	70	19.7	0.94	--
Female	450	73.3			357	58.1			122	19.9		
**Age**	**n**	**%**	**p**	**Post-hoc***	**n**	**%**	**p**	**Post-hoc***	**n**	**%**	**p**	**Post-hoc***
18-24	91	72.2	0.009	35-44 v 55-64	75	60.0	0.67	--	27	21.4	<0.001	25-34 v 65+<br>35-44 v 55-64<br>35-44 v 65+
25-34	131	73.2			100	56.2			41	23.0		
35-44	131	74.9			108	61.7			50	28.6		
45-54	129	72.5			101	56.7			35	19.7		
55-64	90	59.6			95	62.9			22	14.6		
65+	113	62.8			100	55.9			20	11.1		
**Social grade [1]**	**n**	**%**	**p**	**Post-hoc***	**n**	**%**	**p**	**Post-hoc***	**n**	**%**	**p**	**Post-hoc***
A	24	88.9	0.003	--	12	44.4	0.44	--	3	10.7	0.31	--
B	122	70.9			106	61.6			39	22.8		
C1	176	74.3			145	60.9			51	21.4		
C2	140	73.3			103	53.9			28	14.7		
D	86	59.7			82	57.3			26	18.2		
E	96	62.3			93	60.4			35	22.7		
Unknown	40	64.5			39	62.9			13	21.0		

Table S7 (continued) Proportion of participants reporting responses and differences by socio-demographic variables and access to treatment (weighted) (* Post-hoc analyses are shown only for significant pairwise comparisons, p<0.05)

	**Sharing experiences**				**Seeking professional advice and support**				**Making a formal complaint or taking legal action**			
**Employment**	**n**	**%**	**p**	**Post-hoc***	**n**	**%**	**p**	**Post-hoc***	**n**	**%**	**p**	**Post-hoc***
Employed full-time	206	73.3	0.11		156	55.5	0.020	--	64	22.9	0.001	Employed PT v long term illness Economically inactive v long term illness
Employed part-time	83	67.5			61	49.6			15	12.2		
Self-employed	69	69.7			62	62.6			18	18.2		
Economically inactive	186	67.1			162	58.7			44	15.9		
Not working: other reasons	61	77.2			55	69.6			14	17.7		
Not working: disability / long-term illness	76	61.3			83	66.9			39	31.5		
**Education**	**n**	**%**	**p**	**Post-hoc***	**n**	**%**	**p**	**Post-hoc***	**n**	**%**	**p**	**Post-hoc***
GCSE / O-Level	92	67.2	0.038	Bachelor's v No formal qual	75	55.1	0.12		25	18.2	0.009	
Vocational	47	74.6			40	63.5			19	30.2		
A level or equivalent	165	71.7			140	60.9			35	15.2		
Undergraduate degree or equivalent	184	72.2			164	64.6			58	22.8		
Postgraduate degree or equivalent	76	71.0			56	52.3			30	28.0		
Other	48	70.6			39	56.5			11	16.2		
No formal qualifications	70	56.0			64	51.2			17	13.6		

Table S7 (continued). Proportion of participants reporting responses and differences by socio-demographic variables and access to treatment (weighted) (* Post-hoc analyses are shown only for significant pairwise comparisons, p<0.05)

	**Sharing experiences**				**Seeking professional advice and support**				**Making a formal complaint or taking legal action**			
**Geographical region**	**n**	**%**	**p**	**Post-hoc***	**n**	**%**	**p**	**Post-hoc***	**n**	**%**	**p**	**Post-hoc***
East Midlands	43	64.2	0.79		45	66.2	0.82		19	27.9	0.029	North East v North West;
Eastern	60	75.0			45	56.3			13	16.3		
London	102	67.1			87	57.2			35	22.9		
North East	27	67.5			23	57.5			15	37.5		
North West	76	71.0			69	63.9			12	11.2		
Scotland	50	65.8			48	62.3			19	25.0		
South East	112	69.6			91	56.5			32	19.9		
South West	51	64.6			49	61.3			15	18.8		
Wales	46	73.0			32	50.8			11	17.5		
West Midlands	64	68.1			53	56.4			15	16.0		
Yorkshire and Humber	51	77.3			39	58.2			11	16.4		
**Total Impact**	**n**	**%**	**p**	**Post-hoc***	**n**	**%**	**p**	**Post-hoc***	**n**	**%**	**p**	**Post-hoc***
No/mild	122	73.5	0.37		67	41.1	<0.001	No/mild v moderate, no/mild v severe, and moderate v severe	21	12.7	0.002	No/mild v severe
Moderate	251	70.1			198	55.3			66	18.4		
Severe	289	67.7			297	69.7			106	24.8		
**Access / lack of access**	**n**	**%**	**p**	**Post-hoc***	**n**	**%**	**p**	**Post-hoc***	**n**	**%**	**p**	**Post-hoc***
Lack of access	246	68.0	0.315		235	64.9	0.006		44	12.2	<0.001	
Treatment or care	436	71.0			344	56.0			149	24.3		

[1]Social grade: National Readership Survey Classification A (highest grade) to E (lowest grade) https://nrs.co.uk/nrs-print/lifestyle-and-classification-data/social-grade/

Supplementary file: Patient-reported harm from NHS treatment or care, or the lack of access to care

Table S8a: Associations between Sharing experiences and participant characteristics, estimated using logistic regression (n=920)

	N	Shared experiences (%)	Odds ratio	95% CI	p
**Sex**					
Male (reference category)	220	61.8	1		
Female	450	73.3	1.63	1.20 to 2.22	**0.002**
**Age**					
18-24 (reference category)	91	72.2	1		0.061
25-34	131	73.2	1.04	0.58 to 1.88	0.89
35-44	131	74.9	1.23	0.68 to 2.24	0.50
45-54	129	72.5	1.07	0.59 to 1.94	0.83
55-64	90	59.6	0.57	0.32 to 1.02	**0.06**
65+	113	62.8	0.71	0.38 to 1.33	0.29
**Employment** [1]					
Full-time (reference category)	206	73.3	1		0.29
Part-time	83	67.5	0.99	0.58 to 1.68	0.96
Self-employed	69	69.7	0.85	0.49 to 1.48	0.56
Economically inactive	186	67.1	1.07	0.63 to 1.81	0.81
Not working	61	77.2	1.63	0.81 to 3.30	0.17
Disability/ long-term illness	76	61.3	0.69	0.40 to 1.22	0.20
**Education**					
GSCE (reference category)	92	67.2	1		0.77
Vocational	47	74.6	1.17	0.57 to 2.40	0.67
A-level or equivalent	165	71.7	1.02	0.62 to 1.69	0.93
Undergraduate degree or equivalent	184	72.2	1.15	0.69 to 1.90	0.60
Postgraduate degree or equivalent	76	71.0	0.88	0.46 to 1.66	0.69
Other	48	70.6	0.95	0.47 to 1.91	0.88
No formal education	70	56.0	0.74	0.42 to 1.32	0.31
**Social grade 2**					
A (reference category)	24	88.9	1		**0.017**
B	122	70.9	5.60	1.22 to 25.69	**0.027**
C1	176	74.3	1.41	0.71 to 2.80	0.33
C2	140	73.3	1.52	0.79 to 2.94	0.21
D	86	59.7	1.62	0.82 to 3.22	0.17
E	96	62.3	0.77	0.38 to 1.54	0.46
Unknown	40	64.5	0.99	0.50 to 1.96	0.97

Table S8a (continued): Associations between Sharing experiences and participant characteristics, estimated using logistic regression (n=920)

	N	Shared experiences (%)	Odds ratio	95% CI	p
**Region**					
East Midlands (reference category)	43	64.2	1		0.90
Eastern	60	75.0	1.47	0.68 to 3.19	0.33
London	102	67.1	1.19	0.61 to 2.31	0.61
North East	27	67.5	0.97	0.40 to 2.38	0.95
North West	76	71.0	1.53	0.75 to 3.13	0.25
Scotland	50	65.8	1.22	0.58 to 2.56	0.61
South East	112	69.6	1.28	0.67 to 2.46	0.45
South West	51	64.6	1.06	0.50 to 2.24	0.88
Wales	46	73.0	1.95	0.85 to 4.52	0.12
West Midlands	64	68.1	1.27	0.61 to 2.63	0.52
Yorkshire and Humber	51	77.3	1.51	0.67 to 3.39	0.32
**Total Impact**					
No/mild (reference category)	122	73.5	1		0.65
Moderate	251	70.1	0.81	0.52 to 1.26	0.35
Severe	289	67.7	0.86	0.56 to 1.34	0.51
**Harmed through access**					
Harmed through lack of access to care (reference category)	246	68.0	1		0.68
Harmed by NHS treatment or care	436	71.0	1.07	0.78 to 1.46	

[1] Full-time – employed 30+ hours; Part-time – employed 29 hours or less; Economically inactive – full time students, still at school, retired or not in paid work due to long-term illness or disability; Not working – Unemployed and seeking work, Not in paid work for other reasons, or not working – housewife.

[2] Social grade: National Readership Survey Classification A (highest grade) to E (lowest grade) https://nrs.co.uk/nrs-print/lifestyle-and-classification-data/social-grade/

Supplementary file: Patient-reported harm from NHS treatment or care, or the lack of access to care

Table S8b: Associations between Seeking professional support and advice and participant characteristics, estimated using logistic regression (n=887)

	N	Seeking professional support (%)	Odds ratio	95% CI	p
**Sex**					
Male (reference category)	212	59.7	1		
Female	357	58.1	0.89	0.66 to 1.21	0.46
**Age**					
18-24	75	60.0			0.75
25-34	100	56.2	0.66	0.38 to 1.14	0.14
35-44	108	61.7	0.77	0.44 to 1.34	0.35
45-54	101	56.7	0.81	0.46 to 1.41	0.45
55-64	95	62.9	0.85	0.48 to 1.49	0.56
65+	100	55.9	0.73	0.40 to 1.33	0.30
**Employment** [1]					
Full-time (reference category)	156	55.5	1		0.064
Part-time	61	49.6	0.82	0.50 to 1.33	0.41
Self-employed	62	62.6	1.47	0.86 to 2.51	0.16
Economically inactive	162	58.7	1.24	0.75 to 2.04	0.40
Not working	55	69.6	2.27	1.20 to 4.32	**0.012**
Disability/ long-term illness	83	66.9	1.24	0.72 to 2.16	0.44
**Social grade** [2]					
A (reference category)	12	44.4	1		0.310
B	106	61.6	0.40	0.14 to 1.15	**0.09**
C1	145	60.9	1.02	0.51 t 2.05	0.95
C2	103	53.9	0.79	0.40 to 1.54	0.49
D	82	57.3	0.66	0.33 to 1.32	0.24
E	93	60.4	0.68	0.33 to 1.40	0.30
Unknown	39	62.9	0.63	0.31 to 1.28	0.20
**Education**					
GSCE (reference category)	75	55.1	1		0.063
Vocational	40	63.5	1.47	0.74 to 2.91	0.27
A-level or equivalent	140	60.9	1.22	0.76 to 1.97	0.41
Undergraduate degree or equivalent	164	64.6	1.72	1.06 to 2.78	**0.028**
Postgraduate degree or equivalent	56	52.3	1.08	0.59 to 1.97	0.80
Other	39	56.5	0.91	0.47 to 1.76	0.78
No formal education	64	51.2	0.77	0.44 to 1.36	0.37

Supplementary file: Patient-reported harm from NHS treatment or care, or the lack of access to care

Table S8b (continued): Associations between Seeking professional support and advice and participant characteristics, estimated using logistic regression (n=887)

	**N participants**	**Seeking professional support (%)**	**Odds ratio**	**95% CI**	**p**
**Geographical region**					
East Midlands (reference category)	45	66.2	1		0.68
Eastern	45	56.3	0.57	0.27 to 1.21	0.14
London	87	57.2	0.60	0.31 to 1.18	0.14
North East	23	57.5	0.68	0.28 to 1.67	0.40
North West	69	63.9	0.79	0.39 to 1.62	0.52
Scotland	48	62.3	0.71	0.33 to 1.53	0.39
South East	91	56.5	0.58	0.30 to 1.12	0.11
South West	49	61.3	0.56	0.26 to 1.20	0.14
Wales	32	50.8	0.40	0.18 to 0.88	**0.022**
West Midlands	53	56.4	0.61	0.30 to 1.26	0.18
Yorkshire and Humber	39	58.2	0.52	0.24 to 1.13	0.10
**Impact**					
No/mild (reference category)	67	41.1	1		**<0.001**
Moderate	198	55.3	1.80	1.21 to 2.69	**0.004**
Severe	297	69.7	3.82	2.53 to 5.76	**<0.001**
**Harmed through access**					
Harmed through lack of access to care (reference category)	235	64.9	1		
Harmed through NHS treatment or care	344	56.0	0.67	0.50 to 0.91	**0.01**

[1] Full-time – employed 30+ hours; Part-time – employed 29 hours or less; Economically inactive – full time students, still at school, retired or not in paid work due to long-term illness or disability; Not working – Unemployed and seeking work, Not in paid work for other reasons, or not working – housewife.

[2] Social grade: National Readership Survey Classification A (highest grade) to E (lowest grade) https://nrs.co.uk/nrs-print/lifestyle-and-classification-data/social-grade/

Supplementary file: Patient-reported harm from NHS treatment or care, or the lack of access to care

Table S8c: Associations between Formal action and participant characteristics, estimated using logistic regression (n=887)

	N	Formal action (%)	Odds ratio	95% CI	p
**Sex**					
Male (reference category)	70	19.7	1		
Female	122	19.9	0.91	0.63 to 1.31	0.60
**Age**					
18-24 (reference category)	27	21.4	1		**0.018**
25-34	41	23.0	0.82	0.42 to 1.57	0.54
35-44	50	28.6	1.01	0.53 to 1.93	0.99
45-54	35	19.7	0.68	0.34 to 1.33	0.26
55-64	22	14.6	0.47	0.23 to 0.96	**0.038**
65+	20	11.1	0.31	0.14 to 0.69	**0.004**
**Employment** [1]					
Full-time (reference category)	64	22.9	1		**0.09**
Part-time	15	12.2	0.49	0.25 to 0.96	**0.038**
Self-employed	18	18.2	0.88	0.46 to 1.67	0.69
Economically inactive	44	15.9	1.08	0.59 to 1.98	0.79
Not working	14	17.7	0.72	0.33 to 1.57	0.41
Disability/ long-term illness	39	31.5	1.46	0.79 to 2.69	0.23
**Social grade** [2]					
A (reference category)	3	10.7	1		0.71
B	39	22.8	0.31	0.05 to 1.79	0.19
C1	51	21.4	1.19	0.52 to 2.72	0.68
C2	28	14.7	1.14	0.51 to 2.53	0.75
D	26	18.2	0.87	0.38 to 2.02	0.75
E	35	22.7	1.17	0.49 to 2.80	0.72
Unknown	13	21.0	1.07	0.46 to 2.48	0.88
**Education**					
GSCE (reference category)	25	18.2	1		**0.022**
Vocational	19	30.2	2.36	1.08 to 5.13	**0.031**
A-level or equivalent	35	15.2	0.85	0.45 to 1.59	0.61
Undergraduate degree or equivalent	58	22.8	1.85	1.01 to 3.39	**0.045**
Postgraduate degree or equivalent	30	28.0	2.18	1.05 to 4.49	**0.036**
Other	11	16.2	1.09	0.46 to 2.58	0.85
No formal education	17	13.6	1.27	0.59 to 2.73	0.54

Supplementary file: Patient-reported harm from NHS treatment or care, or the lack of access to care

Table S8c (continued): Associations between Formal action and participant characteristics, estimated using logistic regression (n=887)

	N	Formal action (%)	Odds ratio	95% CI	p
**Geographical region**					
East Midlands (reference category)	19	27.9	1		0.16
Eastern	13	16.3	0.58	0.23 to 1.43	0.24
London	35	22.9	0.87	0.42 to 1.82	0.71
North East	15	37.5	1.67	0.66 to 4.24	0.28
North West	12	11.2	0.37	0.15 to 0.89	**0.03**
Scotland	19	25.0	1.05	0.46 to 2.381	0.91
South East	32	19.9	0.73	0.35 to 1.50	0.39
South West	15	18.8	0.84	0.35 to 2.01	0.69
Wales	11	17.5	0.76	0.31 to 1.91	0.56
West Midlands	15	16.0	0.62	0.27 to 1.44	0.26
Yorkshire and Humber	11	16.4	0.52	0.20 to 1.33	0.17
**Impact**					
No/mild (reference category)	21	12.7	1		**0.002**
Moderate	66	18.4	1.61	0.92 to 2.83	**0.01**
Severe	106	24.8	2.55	1.47 to 4.42	**<0.001**
**Access**					
Harmed through lack of access to care (reference category)	44	12.2	1		
Harmed through NHS treatment or care	149	24.3	2.47	1.66 to 3.69	**<0.001**

[1] Full-time – employed 30+ hours; Part-time – employed 29 hours or less; Economically inactive – full time students, still at school, retired or not in paid work due to long-term illness or disability; Not working – Unemployed and seeking work, Not in paid work for other reasons, or not working – housewife.

[2] Social grade: National Readership Survey Classification A (highest grade) to E (lowest grade) https://nrs.co.uk/nrs-print/lifestyle-and-classification-data/social-grade/

Supplementary file: Patient-reported harm from NHS treatment or care, or the lack of access to care

Table S9. Helpfulness of PALS (for n=114 had used PALS) and handling of formal complaint (for n=168 who had made a formal complaint) (weighted)

	Frequency (n)	Percent (%)
**Helpfulness of PALS**		
Very helpful	25	21.5
Fairly helpful	34	29.5
Not very helpful	24	21.2
Not at all helpful	28	24.6
**Handling of formal complaint**		
Very well	17	10.2
Fairly well	33	19.8
Not very well	38	22.8
Not at all well	67	40.2

Table S10. Main reason for not pursuing a legal claim for financial compensation (weighted) (n=962 for those who had not pursued a legal claim)

	Frequency (n)	Percent (%)
Did not want to make a financial claim against the NHS	208	21.6
Did not want financial compensation	175	18.2
Did not occur to respondent	132	13.7
Too stressful or emotionally difficult	91	9.4
Thought it would be too time-consuming	48	5.0
Did not know how to make a claim	46	4.8
Thought it would be too costly	27	2.8
Too unwell	25	2.6
Solicitor/claims company advised against it	15	1.5
Other reason *(offered by respondent[1])*	122	12.7
No reason	58	6.1

[1]Other reasons given included: no point in making a claim; the harm was too mild, and nobody was to blame.

Supplementary file: Patient-reported harm from NHS treatment or care, or the lack of access to care

Table S11a. Variations in responses desired (an apology, an explanation or emotional support) by the NHS following harm by sociodemographic factors and harm through access or lack of access to treatment or care (* post hoc analysis reported for significant results (p<0.05))

		An apology				An explanation				Emotional support			
		Yes				Yes				Yes			
		**n**	**%**	**p**	**Post-hoc***	**n**	**%**	**p**	**Post-hoc***	**n**	**%**	**p**	**Post-hoc***
	**Age**												
**Age**	*18-24*	33	26.2	0.65		43	34.1	0.37		39	31.0	**<0.001**	18-24 v 55-64<br>18-24 v 65+<br>25-34 v 65+<br>45-54 v 65+
	25-34	57	31.8			63	35.2			45	25.3		
	35-44	56	32.0			64	36.6			42	24.0		
	45-54	51	28.7			72	40.4			45	25.3		
	55-64	36	23.8			49	32.5			24	15.9		
	65+	35	19.6			53	29.4			22	12.2		
	Sex												
**Sex**	*Male*	74	20.8	**<0.001**	N/A	106	29.9	**0.017**	N/A	58	16.3	**0.002**	N/A
	Female	188	30.6			230	37.4			152	24.8		
	Employment [2]												
**Employment [2]**	*Employed full-time*	76	27.1	0.08		95	33.8	**<0.001**	PT v not working;<br>PT v disability<br>Econ inactive v not working;<br>Econ inactive v disability	56	20.0	**<0.001**	
	Employed part-time	35	28.5			35	28.2			35	28.5		
	Self-employed	23	23.2			36	36.4			17	17.2		
	Economically inactive	62	22.5			80	28.9			42	15.2		
	Not working	25	31.6			39	49.4			25	31.6		
	Disability / long-term illness	45	36.3			57	46.3			41	33.1		

Table S11a (continued). Variations in responses desired (an apology, an explanation or emotional support) by the NHS following harm by sociodemographic factors and harm through access or lack of access to treatment or care (* post hoc analysis reported for significant results (p<0.05))

	An apology				An explanation				Emotional support			
	Yes				Yes				Yes			
	n	%	p	Post-hoc*	n	%	p	Post-hoc*	n	%	p	Post-hoc*
**Education**												
GCSE / O-Level	31	22.8	0.52	--	46	33.6	**0.003**	A-level v no qualifications	22	16.2	**<0.001**	GCSE v A-level; A-level v Postgraduate
Vocational	20	31.7			27	42.9			17	27.0		
A level or equivalent	70	30.4			102	44.3			74	32.0		
Bachelor's degree or equivalent	72	28.3			86	33.9			54	21.3		
Postgraduate degree or equivalent	29	27.1			33	30.6			15	14.0		
Other	15	22.1			19	27.9			11	16.2		
No formal qualifications	30	24.0			30	24.0			23	18.4		
**Social grade [1]**												
A	5	18.5	0.20	--	8	28.6	**0.003**	B v D<br>C2 v D<br>D v E	4	14.8	0.13	--
B	57	33.1			69	40.1			29	17.0		
C1	68	28.7			82	34.5			59	24.8		
C2	43	22.5			78	40.8			35	18.3		
D	35	24.3			30	20.8			39	27.3		
E	39	25.3			57	36.8			36	23.4		
**Access / lack of access**												
Harmed through lack of access	60	16.6	**<0.001**	N/A	103	28.5	**<0.001**	N/A	73	20.1	0.25	--
Harmed by treatment or care	206	33.6			239	38.9			143	23.3		

[1] Social grade: National Readership Survey Classification A (highest grade) to E (lowest grade) https://nrs.co.uk/nrs-print/lifestyle-and-classification-data/social-grade/

[2] Full-time – employed 30+ hours; Part-time – employed 29 hours or less; Economically inactive – full time students, still at school, retired or not in paid work due to long-term illness or disability; Not working – Unemployed and seeking work, Not in paid work for other reasons, or not working – housewife.

Supplementary file: Patient-reported harm from NHS treatment or care, or the lack of access to care

Table S11b. Variations in responses desired (treatment/care to redress harm; access to treatment previously refused, and staff training) by the NHS following harm by sociodemographic factors and harm through access or lack of access to treatment or care (* post hoc analysis reported for significant results (p<0.05))

	**Treatment/care to redress harm**				**Access to treatment previously refused**				**Staff training**			
	**Yes**				**Yes**				**Yes**			
	**n**	**%**	**p**	**Post-hoc***	**n**	**%**	**p**	**Post-hoc***	**n**	**%**	**p**	**Post-hoc***
**Age**												
18-24	57	45.2	**0.002**	35-44 v 45-54	44	34.9	**0.009**	None	30	23.8	**0.018**	25-34 v 65+
25-34	90	50.3		45-54 v 65+	60	33.7		signif	54	30.3		
35-44	62	35.4			61	34.9			52	29.7		
45-54	97	54.5			55	30.9			49	27.5		
55-64	66	43.7			33	21.9			31	20.5		
65+	67	37.2			39	21.7			30	16.8		
**Sex**												
Male	164	46.2	0.35		107	30.1	0.58		69	19.4	**0.003**	
Female	265	43.1			175	28.5			173	28.1		
**Social grade** [1]												
A	11	39.3	0.61		10	35.7	0.54		0	0	**<0.001**	*A v B*
B	86	50.0			54	31.4			53	30.8		*A v C1*
C1	103	43.5			69	29.0			64	27.0		*A v C2*
C2	79	41.1			58	30.4			51	26.7		*A v E*
D	65	45.5			35	24.5			20	13.9		*B v D*
E	66	42.9			53	34.2			44	28.6		*C1 v D; D v E*
**Access / lack of access**												
Harmed through lack of access	194	53.6	**<0.001**		141	39.0	**<0.001**		68	18.8	**<0.001**	
Harmed by treatment or care	243	39.6			151	24.6			177	28.8		

[1] Social grade: National Readership Survey Classification A (highest grade) to E (lowest grade) https://nrs.co.uk/nrs-print/lifestyle-and-classification-data/social-grade/

Table S11 (continued Variations in responses desired (treatment/care to redress harm; access to treatment previously refused, and staff training) by the NHS following harm by sociodemographic factors and harm through access or lack of access to treatment or (* post hoc analysis reported for significant results (p<0.05))

	**Treatment/care to redress harm**				**Access to treatment previously refused**				**Staff training**			
	**Yes**				**Yes**				**Yes**			
	**n**	**%**	**p**	**Post-hoc***	**n**	**%**	**p**	**Post-hoc***	**n**	**%**	**p**	**Post-hoc***
**Employment [2]**												
Employed full-time	125	44.6	0.09		82	29.2	**0.025**	Econ inactive v not working	79	28.2	0.10	
Employed part-time	61	49.6			35	28.5			25	20.3		
Self-employed	45	45.5			36	36.4			21	21.0		
Economically inactive	106	38.4			70	25.4			59	21.3		
Not working	44	55.7			35	44.3			24	30.4		
Disability / long-term illness	57	46.0			35	28.2			38	30.6		
**Education**												
GCSE / O-Level	62	45.3	**0.024**	A-level v No qualifications	29	21.3	**0.005**	Vocational v No qualifications; Bachelor v No qualifications	33	24.1	0.06	
Vocational	30	47.6			25	39.7			25	39.7		
A level or equivalent	117	50.9			77	33.5			56	24.3		
Bachelor's degree or equivalent	118	46.5			85	33.5			71	28.0		
Postgraduate degree or equivalent	40	37.4			34	31.5			25	23.1		
Other	31	45.6			20	29.4			12	17.6		
No formal qualifications	40	32.0			23	18.4			25	20.0		

[2] Full-time – employed 30+ hours; Part-time – employed 29 hours or less; Economically inactive – full time students, still at school, retired or not in paid work due to long-term illness or disability; Not working – Unemployed and seeking work, Not in paid work for other reasons, or not working – housewife.

Supplementary file: Patient-reported harm from NHS treatment or care, or the lack of access to care

Table S11c. Variations in responses desired (disciplinary action, investigation, financial compensation) by the NHS following harm by sociodemographic factors and harm through access or lack of access to treatment or care (* post hoc analysis reported for significant results (p<0.05))

	Disciplinary action				An investigation				Financial compensation			
	Yes				Yes				Yes			
	n	%	p	Post-hoc*	n	%	p	Post-hoc*	n	%	p	Post-hoc*
**Sex**												
Male	28	7.9	0.07		68	19.1	0.24		30	8.5	0.79	
Female	71	11.6			137	22.3			55	9.0		
**Age**												
18-24	9	7.2	**0.006**	35-44 v 65+	29	23.2	0.42		5	4.0	0.07	
25-34	21	11.7			41	23.0			18	10.1		
35-44	28	16.0			41	23.4			21	12.0		
45-54	18	10.1			39	21.9			18	10.1		
55-64	16	10.6			27	17.9			15	9.9		
65+	7	3.9			29	16.1			9	5.0		
**Social grade** [1]												
A	0	0	Not run		3	10.7	**0.010**	C1 v D<br>D v E	2	7.1	Not run	
B	15	8.8			31	18.0			12	7.0		
C1	22	9.2			58	24.5			16	6.8		
C2	21	11.0			45	23.6			13	6.8		
D	13	9.1			16	11.1			13	9.1		
E	19	12.3			38	24.7			20	13.0		
**Access / lack of access**												
Harmed through the lack of access	16	4.4	**<0.001**		55	15.2	**<0.001**		16	4.4	**<0.001**	
Harmed by treatment or care	83	13.5			152	24.8			69	11.2		

[1] Social grade: National Readership Survey Classification A (highest grade) to E (lowest grade) https://nrs.co.uk/nrs-print/lifestyle-and-classification-data/social-grade/

Table S11c (continued). Variations in responses desired (disciplinary action, investigation, financial compensation) by the NHS following harm by sociodemographic factors and harm through access or lack of access to treatment or care (* post hoc analysis reported for significant results (p<0.05))

	**Disciplinary action**				**An investigation**				**Financial compensation**			
	**Yes**				**Yes**				**Yes**			
	**n**	**%**	**p**	**Post-hoc***	**n**	**%**	**p**	**Post-hoc***	**n**	**%**	**p**	**Post-hoc***
**Employment [2]**												
Employed full-time	25	8.9	**<0.001**	Employed v disability PT v econ inactive PT v disability Econ inactive v not working Econ inactive v disability	58	20.6	**<0.001**	PT v disability Econ inactive v not working Econ inactive v disability	19	6.8	**<0.001**	*FT v disability Econ inactive v not working Econ inactive v disability*
Employed part-time	17	13.8			18	14.6			11	8.9		
Self-employed	9	9.1			20	20.2			9	9.1		
Economically inactive	10	3.6			44	15.9			11	4.0		
Not working	12	15.2			25	31.6			13	16.3		
Disability / long-term illness	26	21.0			40	32.3			22	17.7		
**Education**												
GCSE / O-Level	9	6.6	0.08		25	18.4	0.61		10	7.4	0.33	
Vocational	9	14.3			18	28.6			5	7.9		
A level or equivalent	31	13.5			45	19.6			23	10.0		
Bachelor's degree or equivalent	21	8.3			60	23.6			19	7.5		
Postgraduate degree or equivalent	8	7.5			22	20.6			9	8.4		
Other	3	4.4			13	18.8			2	2.9		
No formal qualifications	16	12.9			24	19.2			16	12.8		

[2] Full-time – employed 30+ hours; Part-time – employed 29 hours or less; Economically inactive – full time students, still at school, retired or not in paid work due to long-term illness or disability; Not working – Unemployed and seeking work, Not in paid work for other reasons, or not working – housewife.
